# Elucidation of Pathways Driving Asthma Pathogenesis: Development of a Systems-Level Analytic Strategy

**DOI:** 10.3389/fimmu.2014.00447

**Published:** 2014-09-23

**Authors:** Michael L. Walker, Kathryn E. Holt, Gary P. Anderson, Shu Mei Teo, Peter D. Sly, Patrick G. Holt, Michael Inouye

**Affiliations:** ^1^Medical Systems Biology, Department of Pathology, The University of Melbourne, Parkville, VIC, Australia; ^2^Department of Biochemistry and Molecular Biology, Bio21 Molecular Science and Biotechnology Institute, The University of Melbourne, Melbourne, VIC, Australia; ^3^Telethon Kids Institute, The University of Western Australia, West Perth, WA, Australia; ^4^Department of Pharmacology and Therapeutics, Lung Health Research Centre, The University of Melbourne, Melbourne, VIC, Australia; ^5^Queensland Children’s Medical Research Institute, The University of Queensland, Brisbane, QLD, Australia; ^6^Medical Systems Biology, Department of Microbiology and Immunology, The University of Melbourne, Parkville, VIC, Australia

**Keywords:** allergy, asthma, systems biology, virus infection, birth cohort, childhood, immune function, epidemiology

## Abstract

Asthma is a genetically complex, chronic lung disease defined clinically as episodic airflow limitation and breathlessness that is at least partially reversible, either spontaneously or in response to therapy. Whereas asthma was rare in the late 1800s and early 1900s, the marked increase in its incidence and prevalence since the 1960s points to substantial gene × environment interactions occurring over a period of years, but these interactions are very poorly understood (1–6). It is widely believed that the majority of asthma begins during childhood and manifests first as intermittent wheeze. However, wheeze is also very common in infancy and only a subset of wheezy children progress to persistent asthma for reasons that are largely obscure. Here, we review the current literature regarding causal pathways leading to early asthma development and chronicity. Given the complex interactions of many risk factors over time eventually leading to apparently multiple asthma phenotypes, we suggest that deeply phenotyped cohort studies combined with sophisticated network models will be required to derive the next generation of biological and clinical insights in asthma pathogenesis.

## Wheezing Diseases in Early Childhood

In common with many chronic diseases, the early stages of asthma development can frequently be recognized during childhood. In particular, the wheezing symptoms that are the hallmark of this disease typically manifest initially during the first 1–3 years of life, and at the population level their frequency is highest in this age group. A range of distinct childhood wheezing phenotypes are now recognized (7), the most important of which are

(i) Transient early wheeze, involving infants with repeated symptoms, which resolve by age ~3 years and recur only infrequently (if at all) thereafter.
(ii) Atopic (allergic) wheeze, in which children express intermittent lower respiratory symptoms associated with sensitization and subsequent exposure to aeroallergens.
(iii) Virus-associated wheeze, in which children experience episodic symptoms in association with respiratory infections but remain wheeze-free at other times.

### Sex differences

There is a clear sex difference in transient early wheeze prevalence during the first years of life, with higher rates observed in boys (8). It is speculated that this is due to smaller airway diameter relative to overall lung size in boys (9). Given equal exposure, this alone would render boys more susceptible than girls to the airway-narrowing effects of the inflammation and associated edema that accompanies activation of host anti-viral defense in the respiratory tract. A similar situation likely applies to allergic airways inflammation, given that aeroallergen sensitization rates are also usually initially higher in boys than in girls (10). With increasing postnatal age these anatomical differences are thought to become much less prominent. This is reflected in the increasingly more uniform rates of wheeze across the genders, with progression through the school years. In general terms, wheezing prevalence at the population level declines after infancy. As discussed below, it is the subset of boys and girls in whom symptoms are maintained that are at highest risk of development of persistent and clinically severe asthma.

## Clinical and Pathological Features of Asthma as it Develops

The general consensus among pediatric respiratory physicians is that, because of the high frequency of transient early wheeze among preschoolers, “asthma” cannot be reliably diagnosed until at least age 5 years in the majority of patients. Affected subjects are characterized by the persistence of their early wheezing symptoms to this age. Similar to the picture gleaned from experimental animal models, the most consistent marker of persistent wheeze is sensitization to aeroallergens, but beyond this comparison the human situation becomes increasingly complex. In particular, while >90% of young asthmatics are atopic, <25% of the overall pediatric atopic population develops asthma despite in many cases virtually continuous aeroallergen exposure [reviewed in Ref. (11)]. By contrast, in the majority of animal models virtually all pre-sensitized animals respond to aerosol challenge via high-level inflammatory responses in the airways, probably reflecting the genetic uniformity of the mouse strains used in these studies, which are pre-selected for responsiveness (12). This suggests that additional mechanisms downstream of allergic sensitization operate in humans that determine (a) the strength of airways inflammation following individual exposure events, and/or (b) the degree to which airways inflammation can be tolerated before it provokes significant clinical symptoms.

A number of innate and adaptive immune mechanisms have been identified as major controllers of the intensity/duration of inflammatory reactions in the airways and a range of evidence suggests that these may malfunction in asthmatics (13–15). Some hints as to the types of additional factors that might be involved can be gleaned from the symptom profiles of children with early stage asthma. A clinical hallmark in these subjects is variable degrees of hypersensitivity to excessively cold, dry, or dusty (particularly irritant laden) air, which provokes cough and/or lower respiratory symptoms, a classic example being “exercise induced wheeze” triggered by the increased ventilatory requirements during energy intensive sports inducing a switch from nasal to oral breathing (16). This is coupled, again to varying degrees among young asthmatics, with exaggerated responsiveness to lower respiratory infections, which frequently triggers wheezing attacks, whereas the same infections elicit milder non-wheezy symptoms in the rest of the population (17).

### Lung function

An important risk factor for persistent asthma is low-lung function, with most longitudinal cohort studies showing a deficit in lung function in asthmatic children when it is first measured (18, 19). In some cases, this may reflect the effects of maternal exposures to environmental toxicants during pregnancy and this is discussed in a subsequent section of this review. However, lung function is also partially under genetic influence and this may contribute to low-lung function in asthmatics (20, 21) and to the familial risk seen in offspring of asthmatic parents. The extent to which genetically determined lung function is altered by postnatal environmental exposures is uncertain. However, as detailed below, longitudinal birth cohort studies have shown that wheezing in early life, especially when associated with respiratory infection, is both a risk factor for subsequent asthma and is associated with lower lung function in childhood (22). The question of the degree to which airway inflammation induced by viruses and other environmental agents is able to damage lungs and further compromise postnatal lung growth has not been fully answered (23), but understanding of the underlying mechanisms is growing, and relevant recent findings are discussed below.

### Airway damage

While histological evidence of airway inflammation and remodeling in infants and young children with recurrent wheeze/asthma is limited, what little evidence there is suggests that recurrent wheeze is associated with thickening of the epithelial reticular basement membrane and airway inflammation (24). These changes seem to relate to the severity of symptoms (24), the need for anti-asthma medication (25) and long-term evidence of airway inflammation (26). However, studies in older children with asthma, cystic fibrosis, and non-cystic fibrosis bronchiectasis show similar histological changes; an increase in smooth muscle mass is observed in all three groups when compared to controls (27), suggesting that these changes may be related to chronic inflammation rather than being disease specific. In addition, recent data from challenge tests in atopic asthmatic adults suggest that bronchoconstriction *per se*, regardless of the mechanism inducing it, increases the secretion of pro-fibrotic cytokines and the deposition of sub-epithelial collagen (28). These factors include TGFbeta, connective tissue growth factor (CTGF), and “TH2” cytokines, most notably IL-13, which is profibrotic, directly and via induced periostin (29–32). The deposition of collagen, a major component of airway remodeling, has been shown to increase airway stiffness and reduce dynamic changes in airway caliber during breathing in adults (33).

### Chronic pathology

Current understanding of how this pathological state is maintained in adult asthmatics is incomplete, but likely involves recurrent cycles of airway tissue injury and repair/remodeling associated with asthma exacerbations, which are triggered by environmental stimuli similar to those discussed below, which are believed to be responsible for initiation of the disease at younger ages. It is increasingly recognized that in adulthood several different asthma phenotypes exist (34–36), some of which are defined via biomarkers indicative of hyper-expression of Th1-, Th2-, and/or Th-17-polarized immunity in airway tissues. How these chronic phenotypes evolve from those observed in children is unknown, but the limited biopsy data available on pediatric asthmatics are consistent with the premise that underlying pathological processes that lead eventually to chronicity are initiated many years before the disease finally consolidates (37), and emerging data from the birth cohort studies are beginning to unravel some of the underlying mechanisms.

## Tracking Wheezing Phenotypes in Early Life: Prospective Birth Cohort Studies

The research strategy that has proven most fruitful in progressively elucidating the initiation phase of asthma involves utilization of large cohorts of children, either randomly selected populations or specific “high-risk” groups recruited on the basis of family history of disease susceptibility. These cohorts are tracked throughout childhood and, where feasible, into early adulthood (38–47). In some instances, exemplified by the “farmer-mother” studies in northern Europe whose offspring express very low susceptibility to asthma/allergy (48), this has additionally involved collecting data on maternal environmental exposures during pregnancy and assessing, the impact of these on subsequent disease risk in their offspring. These cohort studies have involved prospective multidisciplinary/multiparameter phenotypic assessments of subjects across a range of socioeconomic and ethnic groups, in some cases for periods in excess of 20 years, and they are beginning to build a cogent picture of the determinants of asthma susceptibility and resistance.

### Interaction between viral infection and aeroallergen sensitization

Collectively, the two most prominent asthma risk factors identified from the collective birth cohort studies are lower respiratory viral infections and sensitization to perennial aeroallergens [reviewed in Ref. (11, 49)]. In relation to viral pathogens, the principal focus of current research attention on asthma susceptibility is upon rhinovirus infections occurring in the first 2 years of life (23, 40, 50), in particular rhinovirus type C (51, 52). However, respiratory syncytial virus (RSV) has also been identified as a risk factor in this context (40, 53–55), and to a lesser extent parainfluenza, adenovirus, metapneumovirus, and influenza (11), all of which have shown association with symptomatic early infections in cohort studies (40). Viral infections, which occur within the ensuing preschool years also contribute to persistence of wheeze, but the available data are currently insufficient to reliably compute the relative risk for asthma associated with these later infections.

As noted above, symptoms in the majority of children exhibiting virus-associated wheeze during infancy disappear by the early school years (39, 56) by which time they display normal lung function, and these transient wheezers are also usually non-atopic (39). In contrast, children who become sensitized to aeroallergens and who exhibit early infection-associated wheezing symptoms are much more likely to remain symptomatic and have lower lung function by early school age (7, 39, 40, 43). A key issue in this regard is the nature of the relationship between the atopic phenotype and susceptibility to virus-induced wheeze – is it associative or causal?

It has been argued that the atopic state involves intrinsically “unbalanced” immune responses against all forms of environmental antigens, in particular, responses that are skewed toward selective production of cytokines (such as IL-4) that are antagonistic toward development of Th1-dependent sterilizing immunity. There is some support for this in the form of evidence showing diminished and Th2-biased immune responses to rhinovirus by peripheral blood mononuclear cells from adult atopics (57–59), and moreover, innate anti-viral immune functions expressed by airway epithelial cells in established atopic asthmatics may also be defective (60). Thus, it is plausible that immune functions unrelated to allergic sensitization may contribute to increased susceptibility to severe viral infections in subjects with established atopic disease including atopic asthma, but to what extent is unknown.

Emerging evidence from the cohort studies suggests strongly that with respect to disease initiation during early childhood, some of the links between atopy and asthma risk are direct and causal:

1. While early lower respiratory viral infections and development of aeroallergen sensitization are associated with asthma diagnosis by school age, the highest risk is observed in children experiencing both exposures (17, 40, 41, 61) suggesting synergistic interactions between underlying viral- and allergen-triggered inflammatory pathways.
2. For this interaction to occur, it is necessary that aeroallergen sensitization either precedes or is contemporaneous with the relevant viral infections (40, 61, 62).

The level of resultant risk for asthma development is related quantitatively to (a) the level of sensitization achieved during infancy as measured by aeroallergen-specific IgE titres (61), and (b) the intensity of inflammation-associated lower respiratory symptoms expressed during these infections (63). With regard to (b), the relevant asthma-risk-associated infections are those that spread to the lower respiratory tract and trigger symptoms of wheeze and particularly fever (40, 61, 63). Thus, the mechanism by which expression of the atopic phenotype amplifies the asthmatogenic potential of early lower respiratory infections is likely to involve pro-inflammatory effector pathway that is IgE-dependent. The nature of this mechanism is discussed further in a subsequent section of this review.

## Host Factors Influencing Susceptibility to Early Sensitization and Early Infections in Infants

The interim conclusions from the discussion above are that risk for eventual development of asthma by school age is positively associated with concomitant high susceptibility to both aeroallergen sensitization and severe lower respiratory tract infections during infancy. It is well established that the early postnatal period represents the life phase during which resistance to respiratory infections is lowest (64). Similarly, it is now widely recognized that the initiation of allergic sensitization occurs frequently during the first 1–2 years of life, and further that priming of aeroallergen-specific Th2-memory responses during this period can result in allergies that persist into adulthood (11). One of the most detailed pictures of the dynamics of this process has been provided via the Childhood Asthma Study (CAS) cohort in Perth, tracking aeroallergen-specific IgE and associated Th2-memory responses in ~180 subjects at high-genetic risk of allergy/asthma via annual blood samples. This demonstrated initiation of aeroallergen-specific IgE production before age 2 years in virtually all children who attained persistent sensitization status by age 5 years (61).

### Postnatal maturation of the immune system

Heightened susceptibility to infections and allergy in early postnatal life has been linked to the functionally immature state of both the innate and adaptive arms of the immune system at birth (65–67). In the protected *in utero* environment, the fetal immune system is maintained in a relatively quiescent state, and T-helper cell function is subtly skewed to favor production of Th2 cytokines while limiting Th1 response capacity, principally because of the necessity to protect the placenta against the toxic effects of inflammatory cytokines exemplified by IFNγ (68). This form of developmental-associated immune homeostasis is maintained via a multi-layered set of Th2-trophic control mechanisms [reviewed in Ref. (69)], the most direct of which involves transient hypermethylation of the IFNγ promoter in CD4+ Th-cells that are released from the thymus over this period and during early postnatal life (70). It is of interest to note that in contrast, production of IL-17 by Th-cells displays an inverse relationship with developmental age, and is maximal in the neonatal period (71). Th-17 cells are recognized to play a significant protective role in immunity to infections via assumption of Th-1-like effector functions to promote pathogen clearance by enhancement of neutrophil recruitment to infection sites and ensuing activation of macrophages (72, 73); their hyperactivity during early life may represent an evolutionary adaptation to the necessity for transient attenuation of Th1 functions during this period. Innate immune responses in early life are also distinct from those in adulthood. In particular, triggering of most toll-like receptors (TLR) ligands in neonatal leukocyte populations yields less IL-12, type 1 IFN, and TNFα, but enhanced levels of IL-1, IL-6, IL-23, and IL-10 relative to corresponding adult cells; overall, the neonatal innate response profile supports robust Th-2 and Th-17 immunity while Th-1 immunity is attenuated (74).

Survival in the microbially hostile extra-uterine environment necessitates upregulation of both innate and adaptive immune response capacity, in particular Th1-associated functions, as the effectiveness of short-term protective humoral mechanisms operative during the neonatal period – such as transplacentally transferred IgG and allied immunoactive factors provided in colostrum and milk, and hyperactivity of endogenous Th-17 cells – progressively wane with age. It is clear that the kinetics of the overall postnatal immune maturation process is highly variable across the human population, and within individuals the rates of maturation in different effector and regulatory pathways are rarely synchronous. Of particular interest are observations linking delayed postnatal maturation of certain key immune functions with increased susceptibility to subsequent development of atopic and/or asthma-associated phenotypes. This has been demonstrated both in prospective studies tracking cohorts of children through to disease outcomes, and in cross sectional studies comparing groups of children at low versus high-genetic risk of atopy/asthma, prior to disease onset. Immunological parameters, which have been implicated in this context include IFNγ response capacity (75–77), IL-12 production (78, 79), HLA-DR expression (80), and the numbers/functions of T-regulatory cells (81) and dendritic cells (DC) (82–84). Increased susceptibility to severe lower respiratory tract infections during infancy has likewise been linked with developmental deficiency in the circulating DC compartment (84), with decreased capacity for production of IFNγ (85) and/or IL-12 (86, 87), and also with an imbalance between production of pro-inflammatory and regulatory cytokines by circulating Th-cells (88). Moreover, young children at high-risk of atopic diseases additionally display attenuated responses to both DTaP (89) and pneumococcal polysaccharide vaccine (90).

### Variability in early immune development

Collectively, these findings suggest that susceptibility to early infections, allergic sensitization, and subsequent asthma are linked to a common set of subtle developmental deficiencies related to postnatal maturation of innate and adaptive immune functions. It is important to note that the available data indicate that these deficiencies, in particular, those related to postnatal expression of Th1-associated functions, are transient and typically resolve after infancy (89). Of additional interest, in many cases the children exhibiting this at-risk immunophenotype display a prominent “rebound” in immunocompetence by the early preschool years and become hyper producers of both Th1 and Th2 cytokines in Th-memory responses (69, 89, 91), and it appears possible that this heightened immunoactivity state may contribute to subsequent symptom expression once asthma becomes consolidated (92, 93)_._ However, the short-term consequences of these transient developmental deficiencies may be even more important in relation to subsequent asthma pathogenesis, and this will be discussed further below.

## Impact of Environmental Factors: The “Hygiene Hypothesis,” Postnatal Maturation of Immune Function, and the Microbiota

The attention of the allergy/asthma research community to the potential role of environmental microbial factors as determinants of disease susceptibility was first raised via publication of the “hygiene hypothesis” by Strachan in 1989 (94). In the original iteration of this theory, high rates of infections in children from large families were proposed to protect against allergy development by some form of “bystander” immunostimulation. However, this concept has evolved considerably over time. As noted above, the transition from the relatively microbial-free maternal environment to the outside world necessitates radical changes in both the “polarity” and overall activity state of the immune system. This includes but is not restricted to reversal of the Th2/Th1 balance that characterizes the neonatal immune system toward a more Th1-dominant state to facilitate programing of efficient Th1-dependent sterilizing immunity, at the same time increasing resistance to development of the excessively Th2-polarized memory responses to non-pathogenic antigens that are associated with allergy.

### Human microbiome

A broad range of evidence suggests that postnatal maturation of immune function in mammals is driven by environmental microbial derived stimuli, and the strongest signals appear to come from the commensal microbiota of the gastrointestinal tract [reviewed in Ref. (95)], the seeding of which is normally initiated during the birth process (96). Exemplary of this relationship, immune function in mice maintained in a gnotobiotic (germ-free) state postnatally remains highly Th2 polarized, and they are unable to develop protective immunological tolerance to exogenous non-pathogenic antigens and instead default to allergy-associated Th2-dependent IgE responses following exposure (97). It is pertinent to note in this context the epidemiological evidence indicating that cesarean delivery in humans is associated with increased risk both for subsequent allergy development (98–100) and for severe lower respiratory tract infections during infancy (101), and it is widely speculated that this may be the result of differing patterns of gut bacterial colonization in the immediate postnatal period relative to newborns delivered vaginally.

### Airborne microbes

It is additionally of interest to note a series of recent findings relating to northern European “farmer mothers” and their offspring, who are heavily exposed to airborne microbial stimuli (exemplified by bacterial lipopolysaccharide) in dust from animal-holding barns in which the mothers work (including during pregnancy) for several hours per day (48). Earlier studies have shown that postnatal exposure to lipopolysaccharide in household dust is associated with a moderate but significant reduction in risk for development of allergy in early childhood (102). However, the tracking of postnatal asthma and allergy development in the offspring of the heavily exposed farmer mothers has provided strong evidence [reviewed in Ref. (48)] that the offspring of these mothers develop strikingly higher levels of resistance to both asthma and allergy relative to the household dust-exposed populations. Furthermore, the development of resistance was independently associated with exposure of the children themselves to barn dust postnatally, and with exposure of their mothers during pregnancy, the latter, resulting in more robust immunoregulatory function in their newborns (103). Follow-up animal studies pinpointed the mechanism underlying this maternal effect on immune development in their offspring as low-intensity innate immune responses to microbial products in inhaled barn dust, which result in indirect attenuation of potentially pro-inflammatory TLR functions in the maternal decidua (103), likely operating via a bone marrow-placental axis (104). It is speculated that this quiescent state at the feto-maternal interface is protective toward placental function, thus, promoting optimization of the growth and development of the fetal immune system, resulting in higher levels of immunocompetence (and hence disease resistance) in the offspring at birth (104, 105).

## Development of Persistent Asthma: The “Critical Window” Concept

In common with the immune system, postnatal survival dictates that the respiratory system of newborns must rapidly adapt to the demands placed on it by the outside world, in particular, it needs to establish response thresholds to inhaled stimuli that will enable it to “tolerate” normal levels of exposure to ubiquitous environmental irritants. This adaptation includes the gross structural changes associated with physical growth, which *inter alia* progressively increases airway diameter, thus, reducing susceptibility to the physical obstruction that frequently accompanies episodic airways inflammation in infants. A range of more subtle processes are involved in parallel including alveolarization and accompanying changes in the airway epithelium, which continue beyond infancy. Local growth and differentiation of non-adrenergic non-cholinergic nerves also establishes neural control of irritant receptor systems and airway smooth muscle during this period (106). These pathways are particularly prominent in rodent animal model where both inhibitory and excitatory NANC pathways have strong effects on airway caliber and antidromic activation of neuropeptideric excitatory NANC pathways induces neurogenic inflammation (107).

Parenchymal lung tissues undergo comparably profound changes in this early postnatal period, and any aberrations in normal growth patterns in this compartment, which result in abnormalities in parenchymal lung mechanics in infants, are postulated to contribute to the lung function changes that are part of the persistent asthma phenotype in later life (108). More direct support for the general concept that structure–function changes initiated during this early postnatal phase of rapid growth can exert long-term effects on the respiratory system comes from studies in infants that demonstrate stable “tracking” of lung function over the course of early childhood, i.e., while lung function progressively increases in all subjects in absolute terms as they mature, the relative position of individuals within the overall population distribution tends to remain close to where they were at birth (109).

### Long-term impacts of events in the early postnatal period

**Figure 1:**
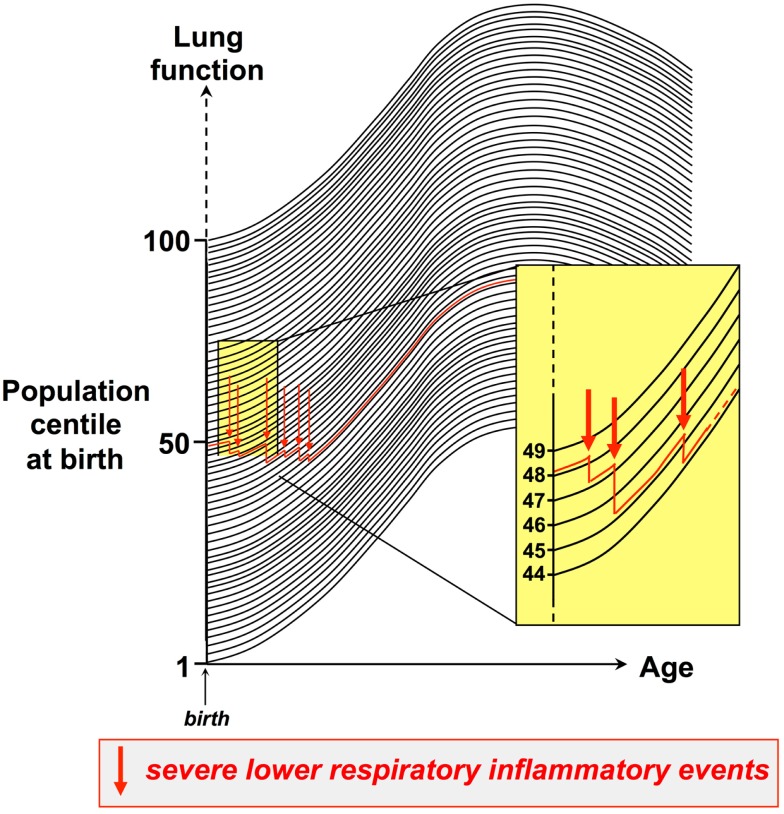
**Establishing trajectories for maturation of respiratory function during childhood**. Respiratory function can be expressed as population centiles, and during the preschool years individual children typically track on the same respiratory function centile (dark lines) defined by their personal centile at birth. Experiencing recurrent inflammatory events of sufficient intensity and duration to perturb ongoing growth/differentiation of lung tissues can result in non-reversible tissue remodeling, accompanied by stepwise dropping to progressively lower levels of lung function. The figure exemplifies an infant who is in the 49th centile at birth, who after three such severe events has declined to the 44th centile.

Moreover, the available evidence suggests that this tracking phenomenon also applies to the sequelae of events associated with respiratory stress or trauma to the respiratory system, particularly severe lower respiratory tract viral infections, which can interrupt normal lung growth and result in a drop to a lower centile within the respiratory function population distribution, which is then maintained. An archetypal example is RSV infection in infancy, which is a major risk factor for wheeze by the end of the preschool years, and this is associated with persistence of low-baseline lung function that is correctable by bronchodilator (56), implying that the early infection can result in increased airway smooth muscle tone, which tracks for years thereafter. This process is illustrated in Figure 1. Thus, respiratory function can be expressed as population centiles (dark lines in Figure 1), and during the preschool years individual children typically track on the same level defined by their personal centile at birth. Experiencing recurrent inflammatory events of sufficient intensity and duration to perturb ongoing growth/differentiation of lung tissues can result in non-reversible tissue remodeling, accompanied by stepwise dropping to progressively lower levels of lung function. The example illustrated (red line in Figure 1) exemplifies an infant who is in the 49th centile at birth, who after three such severe events has declined to the 44th centile. In principle, this down-stepping process can continue over a period of years, but the impact of individual inflammatory episodes is likely inversely related to the postnatal age at which they occur.

Additional studies have shown that respiratory function also tracks from childhood into adulthood (110, 111). This finding is very consistent with long-term asthma cohort studies, which indicated that for the vast majority of patients, the degree of severity they acquire as children will remain a constant in their lives (111). Very few patients show progressive worsening of disease when assessed on a population basis.

### Antenatal versus postnatal factors

**Figure 2:**
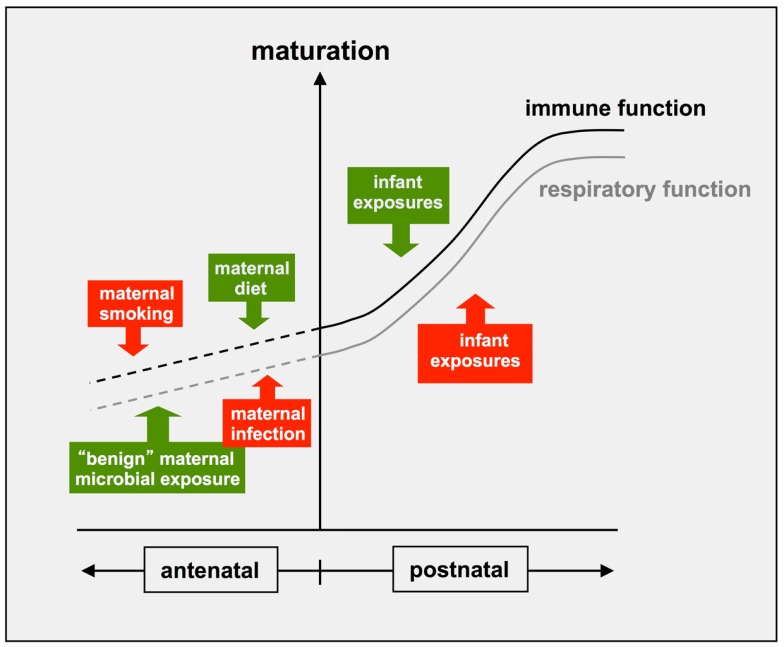
**Antenatal and postnatal factors influencing development if immune and respiratory functions**. Recent evidence indicates that the postnatal maturation of both respiratory and immune functions can be influenced by positive (green) and negative (red) environmental influences, which can exert their effects after birth and/or antenatally.

There is additionally growing evidence that similar processes can occur at earlier stages of development, as illustrated in Figure 2. In particular, dietary factors, which are likely to include microbial products in unpasteurized milk, have been identified as an important component of the “asthma/allergy-protective” signal present in the environment of the northern European farmer mothers (48, 112). Maternal environmental exposures can also impact negatively on these developmental processes. A well documented example relates to the offspring of mothers who smoke during pregnancy: these infants typically have reduced lung function at birth relative to the overall population; this reduced function tracks into adolescence (113–116) and is associated with persistent wheeze and asthma (117, 118). Maternal smoking during pregnancy is also a risk factor for asthma persisting into adolescence, independent of effects on lung function and immune-phenotype (110). It is important to note that the same general principles apply to the immune system, which also displays dynamic maturational changes during fetal development, which are further refined postnatally (Figure 2). In this regard, the offspring of the smoking mothers also show altered patterns of cytokine production at birth and associated increased risk for postnatal allergic sensitization (118), and reduced numbers of circulating T-regulatory cells (119), indicating effects on early immune development. Of particular note, the protective “farmer-mother” environment described above promotes reciprocal effects on T-regulatory cells in their asthma-resistant offspring (100).

Maternal exposure to indoor air pollution especially that is associated with biomass fuel use for cooking and heating, in developing countries is a particular problem and associated with both acute and chronic respiratory diseases in the offspring (120, 121). Maternal infections during pregnancy, particularly those associated with antibiotic use, have also been associated with increased propensity for later wheeze in offspring (122, 123). Thus, the temporal window period during which functional programing of the key physiological processes underpinning subsequent risk for asthma spans both the antenatal and early postnatal periods.

## Progression toward Persistent Asthma

A diagnosis of asthma at the end of the preschool years implies increased risk for progression to persistent asthma in the teens and beyond, but at the population level only 50–60% of early-onset asthmatics follow this track, and at least through to the end of the teenage years the majority of the latter are atopics (93, 124). In these subjects, cumulative pathological changes resulting from episodic cycles of airway tissue damage and ensuing repair/remodeling continue to drive pathogenesis, progressively eroding levels of respiratory function relative to their non-asthmatic counterparts (11, 49). The most overt manifestations of this process are asthma exacerbation events, particularly those occasioning physician visits, and the decrement in ensuing lung function after each episode reflects their intensity and duration (125). It is now well established that as per the initiation phase of asthma in preschoolers, the principal environmental triggers of asthma exacerbations in older children and adults with established disease are aeroallergens and respiratory viral infections, acting independently and/or in synergy (11, 49) (Box 1).

**Box 1:**
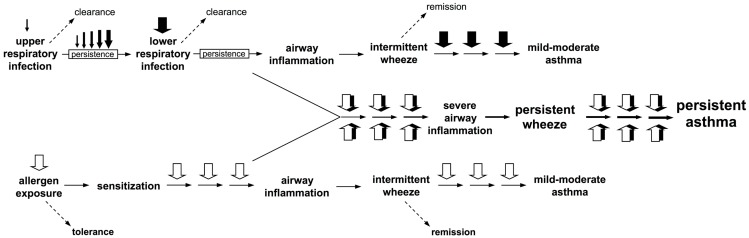
**Interactions between atopic and anti-viral inflammatory pathways during exacerbations drive progression to persistent asthma**. Two clear pathways to wheeze development are now recognized. Firstly, upper respiratory viral infections which evade clearance, and instead persist and intensify and spread to the small airways of the lower respiratory tract where they trigger intermittent inflammatory responses and associated wheeze. These symptoms frequently remit, but recurrence of such episodes can result in their persistence and this is associated with heightened risk for asthma development. Secondly, sensitization to perennial (especially indoor) allergens which are present continuously in the domestic environment can also lead to airway inflammation of generally lower intensity than those associated with infections, but such events are likely to occur considerably more frequently, and can also be associated with asthma risk. However, the highest risk is associated with concomitant exposure to virus and aeroallergen against a background of aeroallergen sensitization, resulting in interaction(s) between the pro-inflammatory pathways triggered by these agents that appear synergistic.

Recent studies from our group and others have elucidated a series of interrelated mechanisms that underlie interactions between viral and allergen-triggered inflammatory pathways [reviewed in Ref. (49)]. The key mechanism involves viral triggered upregulation of FcER1-expression on local airway mucosal (AM) DC that results in recruitment of “bystander” aeroallergen-specific Th2-memory responses into the inflammatory milieu at the infection site, which in turn attenuates Th1-dependent anti-viral defense mechanisms and delays viral clearance. Ensuing type 1 IFN- and IL-4/IL-13 signaling to the bone marrow results in upregulation of FcER1-expression on DC precursors prior to their release into the circulation for migration to the infected airway mucosa to replenish the rapidly turning over AMDC population. This further enhances local aeroallergen-specific Th2-memory cell activation, amplifying inflammation, and further retarding viral clearance. Consistent with this sequence, gene co-expression network analysis of sputum cells collected from exacerbating children indicated expression of Th2-associated effector genes in conjunction with attenuated expression of Th-1/cytotoxic pathway genes, and linked the level of this attenuation with the degree of chronic airflow obstruction, which followed the event (126).

It is important to note that this interaction between atopy/viral infection appears operative across the full spectrum of virus-associated wheezing events including those in mild asthmatics (17). Moreover, IL-4/IL-13 signaling from sites of allergy-induced inflammation to the bone marrow clearly occurs in the absence of viral infection, resulting in upregulation of FcER1-expression in the circulating DC precursor pool that is likely related to the intensity/duration of the inducing event (49). The same precursor pool continuously replenishes mucosal DC populations throughout the body, and this process hence provides a putative mechanism through which allergic manifestations in one tissue can promote the spread of allergic disease to other (previously unaffected) tissues by enhancing the baseline Th2-stimulatory potential of DCs in the latter (49). Such cross-tissue relationships have long been recognized in the allergy field, exemplified in this context by the epidemiological data linking allergic rhinitis in young adults with risk for subsequent development of asthma (127, 128). The “Allergic March” concept in pediatric allergy in which food allergy during infancy precedes rhinitis, which in turn precedes asthma [reviewed in Ref. (49)] would appear to represent another example of this process in action.

## Additional Complexities in Asthma Development

The pathways illustrated in Figures 1 and 2 and Box 1 provide plausible mechanistic explanations for some of the major epidemiological associations observed in relation to risk for asthma development, but cannot accommodate important emerging data from other crucial areas.

### Genetic susceptibility to disease

Susceptibility to asthma is recognized to be in part genetically determined and, while asthma heritability is less than many other complex diseases, to date variants in excess of 100 genes have been identified as potential contributors to this risk (1, 3, 4, 6). Moreover, *specific gene* × *environment* interactions exemplified by those involving microbial exposures and asthma/allergy-related outcomes (2, 5, 129–131) are increasingly being identified in the context.

### Childhood obesity

Obesity is increasingly prevalent in children and there is increasing evidence that obesity affects childhood asthma and its severity (132). The nature of the association between obesity and asthma remains obscure in children and appears to be stronger for non-atopic asthma. Longitudinal studies suggest that high body weight precedes asthma symptoms. However, it is not clear how body mass may influence primary acquisition of persistent wheeze. It is also of interest that body mass can exert cross generational epigenetic effects so it is possible that parental obesity may affect their offspring.

### Airway microbiota

Until very recently, it was assumed that at baseline the lower airways of normal immunocompetent individuals is maintained in a sterile condition, punctuated by only occasional incursions in the form of viral infections accompanied in some instances by opportunistic bacterial pathogens derived mainly from the nasopharyngeal flora. However, a recent study using bacterial metagenomics and applied in conjunction with sterile fiber-optic bronchoscopic sampling of the lower airways has demonstrated the presence of complex bacterial flora extending from the nasopharynx to the alveolar spaces in healthy individuals (133). There is emerging evidence that in diseases such as asthma and COPD the density and complexity of this airway flora may be increased relative to the overall population, but the currently available data must be considered principally qualitative rather than quantitative and reflects the very early stage of research in this area.

We have recently reviewed the theoretical implications of these new findings (134). Notably, data exemplified by the CAS birth cohort (61) demonstrate that it is common for infants within the high-risk “window period” for asthma development to experience three to six moderate–severe lower respiratory viral infections per year, and it must now be accepted that virtually every one of these episodes will involve some level of breaching of airway epithelial barriers by “bystander” bacterial flora, which are present in fluids overlying the airway mucosa at the time of viral infection. As noted (134), this carries the attendant risk of bacterial-induced amplification of local inflammatory tissue damage at the viral infection site, and/or potential secondary bacterial infection as pneumonia or sepsis, unless clearance is rapid. In this regard, it is of interest that early colonization of the nasopharynx with bacterial pathogens has been linked with increased risk for subsequent development of asthma in preschoolers (135). It is therefore relevant to question whether variations in antibacterial host defense mechanisms may also contribute to asthma susceptibility in young children. Preliminary evidence from the CAS birth cohort (134, 136) suggests that the efficiency of immune surveillance for common nasopharyngeal bacterial species during infancy as measured via specific IgG1 antibody titers is inversely related to risk for subsequent development of atopic asthma in children, and this may reflect the role of this antibody in bacterial clearance. Moreover, we have recently reported that Th2-polarized immunity in school children against these bacterial species is protective against expression of asthma symptoms (137). The surrogate marker for this latter effect is IL-4/IL-13-dependent IgE antibody against bacterial-specific particulate antigens, but we theorize that protection is mediated by bacterial-specific IL-4/IL-13-producing Th2-memory cells that employ these same cytokines to inhibit bacterial-induced activation of airway tissue macrophages at sites of low-level bacterial incursions (134, 137).

These new findings have collectively opened, yet another chapter in the already highly complex story of the immunopathogenesis of human asthma. It is clear that high-precision experimental models, which focus on individual inflammatory effector pathways can provide invaluable understanding relating to their targeted mechanisms, but applying this information to the development of treatment strategies that must account for variations in patient age, disease stage, and in particular, disease sub-phenotype has proven to be a major and still outstanding challenge (49).

### Need for complex models

Table 1 summarizes the multiple known and presumptive risk factors for the development of asthma discussed above. Given the breadth and diversity of these factors and the relative long-time frame over which their effects may be exerted, the relevance of cohort studies where objective longitudinal data collected over decades becomes immediately clear. One of the most important recent innovations has been the application of mathematical modeling methods to cohort data in an effort to parse out the important interactions and driving mechanism leading to persistent asthma.

**Table 1:** **Known and presumptive factors though to influence the development and persistence of asthma during early childhood**.

Known or suspected determining factors	Strength/direction
Wheezing viral infection	+++
Febrile viral infection	+++
Sensitization to perennial aeroallergens	+++
Sensitization to other classes of allergens	++
Age of infection and type of virus	++
Genetic predisposition to Th2 immune bias	++
Daycare	+
Sex	+
Maternal smoking	+
Postnatal tobacco smoke exposure	+
Maternal and antenatal infection (and antibiotic use)	+
Bronchial hyperresponsiveness	+
Low-lung function/lung growth	+
Mucosal inflammation	+
Obesity	+
Lung microbiota	+
Cesarean delivery	+
Maternal and antenatal environmental exposure	++/−−
Domestic animal exposure	+/−
Microbiota development in gut	+/−
Maternal and antenatal nutrition	+/−
Number of siblings	−

*Once identified, such factors can be measured and the data introduced into modeling systems to generate and test hypotheses on asthma development*.

*“+” contributes to etiology/pathogenesis and “−” protective*.

## Modeling Birth Cohorts Using Networks

A useful way of thinking about human development in infancy and childhood is that of a (spatio)temporally dynamic network of interacting systems. The multi-factorial nature of asthma pathogenesis implies that many such systems need to be considered, including the respiratory, immune, and microbial systems. These systems are constrained by the genomes of their respective organisms and are impacted by environmental factors, including viral infections and inhaled exposures.

An accurate and robust network model of the factors comprising asthma pathogenesis may be effective in identifying and prioritizing prevention and intervention strategies at the population and individual level. Indeed, such an approach has already been advocated for modeling the immune system (138). Deeply phenotyped cohort studies, such as CAS (40, 139), MAS (39, 140), and URECA (141), will be critical in the building and validation of such network models. These studies present a broad range of detailed data with the corresponding time order, allowing researchers to map the paths of disease pathogenesis and gain insight into causation, prevention, and possible interventions. Further, network modeling of such cohorts would take advantage of the wealth of expert knowledge in asthma pathogenesis, such as that in prior sections of this review.

### Network models

The behaviors of biological networks can be complex. For this reason, as well as a human predilection for pairwise relationships, network models are still rarely the first approach for many researchers. Yet, tools for studying networks have so far found a diverse range of applications including the imputation of missing data from an asthma intervention/education program (142), the relationship between ozone point data and regional asthma (143), the role of cytokine networks in asthma (144) and the identification and characterization of transcriptional networks for IgE signaling (145, 146). Further, with the increasing utilization and integration of genomic and other omics profiling, a network modeling framework offers a powerful approach for extracting maximum biological information for massive datasets (147).

**Figure 3:**
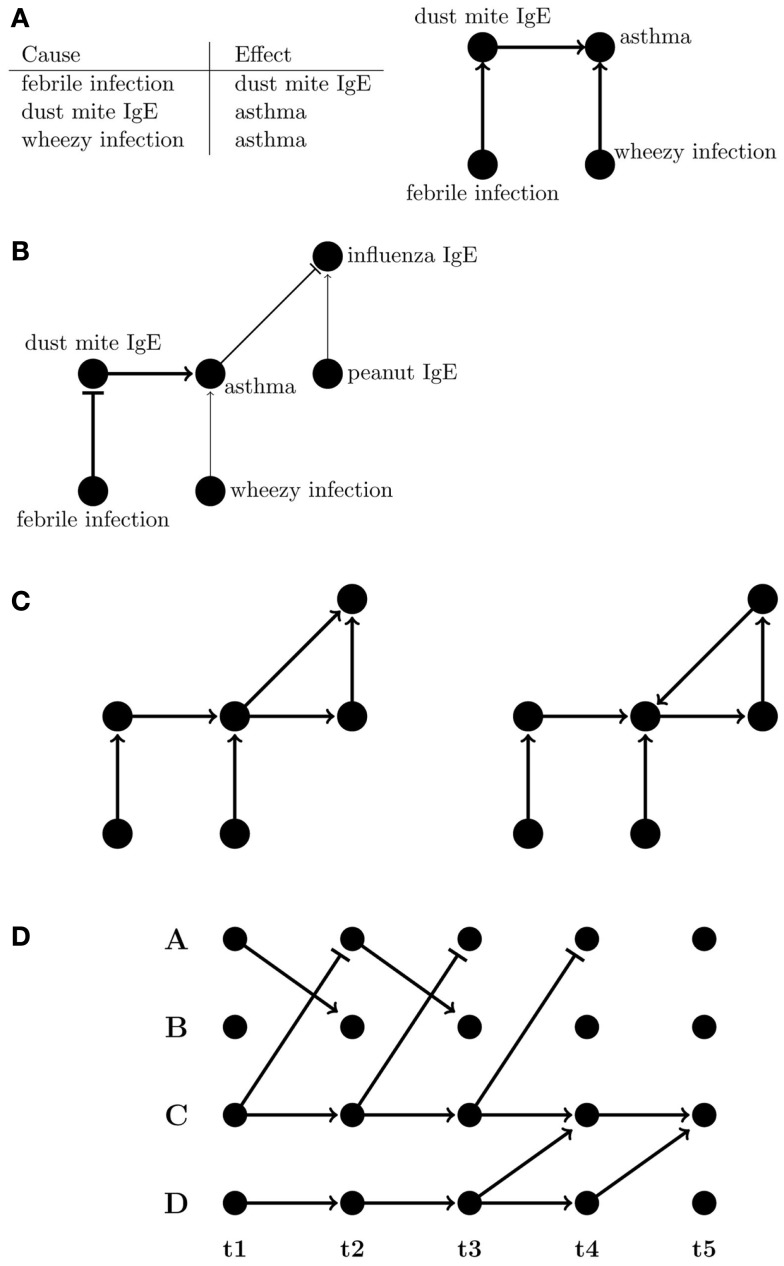
**Representations of directed networks**. **(A)** Tabular and graphical (left and right, respectively) displays of directional flow between variables. **(B)** Graph showing variations in strength and sign of effects between variables. The flattened edges indicate negative relationships. **(C)** Directed acyclic (DAG) and directed cyclic graphs (left and right, respectively). **(D)** A dynamic Bayesian network where edges are only between subsequent time points.

A network graph is a versatile means of representing the multiplicity of relationships that can exist, whether they are simple correlations or of a directed, often causal, nature. Graphs of networks, comprising nodes (representing entities such as phenotypes), and connecting lines called “edges” (e.g., representing relationships between phenotypes), can be an intuitive way of capturing complex interactions; for example, most would find the graph rather than the table in Figure 3A easier to comprehend, and graphs can also visually indicate the strength and sign of relationships between nodes (Figure 3B).

### Bayesian network models

A popular and mathematically rigorous example of a network, which seeks to model causal relationships, is a Bayesian network (BN), a type of directed acyclic graph (DAG) (Figure 3C). BNs are particularly useful for biological and clinical data because they are inherently probabilistic, tolerant of incomplete or missing data, and there are wide array of robust tools available to researchers wishing to use them. Importantly, they explicitly allow for integration of prior expert knowledge into the model. BNs have also been applied to biological and clinical problems such as diagnostics (148), cell signaling pathways (149, 150), asthma exacerbations in a pediatric emergency department (151), and transcriptional networks associated with asthma susceptibility (152). For cohort studies, a useful feature of BNs is that mutual interactions and self-interactions can still be represented if the factors involved have corresponding nodes at multiple time points. Longitudinal data allow for better inference of causal relationships, allowing one to infer the relationship between node A at time *t*_i_ and node B at subsequent time *t*_i + 1_ even if A and B are the same phenotype. So-called “un-rolled” BNs, i.e., those that allow their topology to change over time, are referred to as dynamic Bayesian networks (DBNs) (153–157) (Figure 3D). While homeostatic self-regulating systems like immune networks can have multiple cyclical feedbacks, DBNs, and other models for how multiple susceptibilities and risks affect disease over time can account for cyclic feedback by making the assumption that time itself always moves forward. Events in the future do not influence events in the present or past.

### Model inference

While an expert can manually construct networks from known relationships, efficient algorithms can infer them from raw data (158–163). Many are of a “hill-climbing” variety (164), in which a graph/network is varied in some way, usually (semi-) randomly, evaluated for its fit to the data and accepted if the resulting network is found to be an improvement. Repeating this process, the algorithm finds a succession of networks of increasingly better fit to the data. To avoid getting “stuck” on a suboptimal network, which happens to be better than those most similar to it, it is possible to run the algorithm multiple times from different starting points (156) or to allow (165) limited excursions “downhill” so that higher hills (which better fit the data) might be found.

Another generic approach is to infer appropriate networks using Bayesian inference (166–174). The operating principle for Bayesian inference may be reasonably characterized as saying that a network is more likely to be correct if it is more likely to generate the data. These so-called *marginal likelihoods* are weighted by any information already available, which might indicate the true values. This ability to incorporate *prior* information can be a major advantage of the Bayesian approach (166, 170). Of particular relevance to clinical application, Bayesian inference has long been utilized for classification and prediction (175–177). Bayesian classification has been applied to such quality of care assessment for hospitals (178) prediction of outcomes of medical procedures (158, 178–180), and the association of genetic data with phenotypes (181).

### Naïve Bayesian networks

The simplest BN, successfully used for many classification and prediction applications (175–177, 182–184), assumes that the probability distributions of the variables of interest are independent. While this assumption is clearly strong for a complex disease such as asthma and the deeply phenotyped cohorts needed for its study, the so-called *naïve* Bayes model is still instructive as a basic BN, is relatively accessible as a model for clinical and biological researchers, and can be illustrative of the effects, or lack thereof, of modeling assumptions. Indeed, the *naïve* Bayes model can be more accurate as a classifier than one might reasonably expect. While violations of the independence assumption can be detrimental to the algorithm (185), it has often been observed that *naïve* Bayes performs comparably to more sophisticated approaches with fewer assumptions (183). Yet, more sophisticated approaches that, for example, estimate joint, conditional and marginal probability distributions from the data are usually not solved exactly due to computational limitations so approximation methods must be used to infer the network (186–191).

## Comparison of a Simple (Naïve) Bayesian Network to Logistic Regression Using the Perth-Based Childhood Asthma Study

To demonstrate the utility of a simple BN on cohort data, we performed a comparison of the performance of the *naïve* Bayes and logistic regression models. Performance in this case has been defined as prediction of a child’s wheezing phenotype at 5 years of age without regard to network topology (as the true network is not known) and we caution that the performance in this case is not a fully realized prediction model, and as such is suitable for method comparison only. In this case, *naïve* Bayes simplifies the problem of BN inference and illustrates the advantage of the prior. To perform the analyses, we utilized the open-source R statistical package *klaR* (192) and the *glm* function in *stats*. Since asthma and its characteristic wheezing phenotype are complex multi-factorial diseases whose pathogenesis often begins in childhood, we simultaneously model a wide cross-section of 45 CAS phenotypes collected in the first 2 years of life (Table S1 in Supplementary Material). These include measures of respiratory infection, including virus-specific confirmation, circulating antibody profiles, and environmental variables, as well as previously published risk factors (40, 61). For simplicity, we assume that all variables are Gaussian and each prior is set to the mean across all the data.

### Model construction

Construction of the models proceeded via cross-validation. We used two-thirds of the CAS data to train both logistic regression and *naïve* Bayes models to predict wheeze status at 5 years given data. The resulting models were then externally tested on the remaining third of the data (the “test” set) to determine their performance in predicting subsequent wheeze. We minimized the effect of stochasticity from sampling variation by repeating this process 100 times for each model, taking different training and testing datasets each time, then averaging the result. We also ensured that the proportion of wheezing individuals in both training and testing datasets reflected that of the overall study and missing data was replaced with corresponding mean values.

### Performance

**Figure 4:**
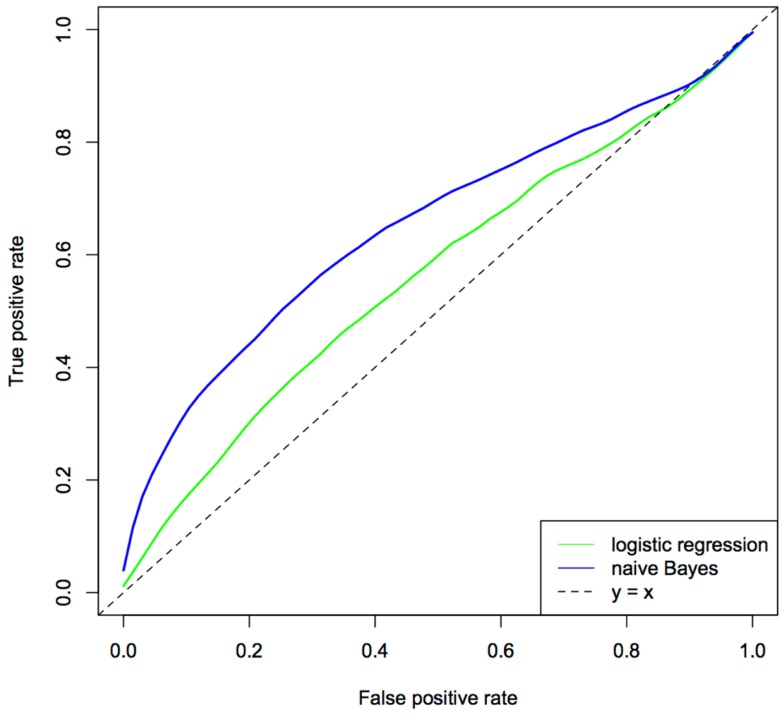
**Comparison of logistical regression and naïve Bayes ROC curves**. The predictor set contains 45 different predictors, including measures of respiratory infections, immune function, lung function, and maternal and environmental factors. The ROC curve was generated using 100 × 3-fold cross-validation.

The relative performance of the two approaches was assessed using the area under the ROC curve (AUC), a measure, which considers both sensitivity and specificity and can be interpreted as the probability of a given model correctly ranking a disease case higher than a healthy individual. The ROC curves for logistic regression and *naïve* Bayes models are given in Figure 4. From these analyses, the *naïve* Bayes was the better predictor (i.e., explained more variance in the wheeze phenotype) with an AUC of 0.64 compared to an AUC of 0.57 for logistic regression. These results have implications for modern day cohort studies where the number of phenotypes, especially those generated from omics technologies, will usually be greater than the study’s sample size and, further, inevitably there will be a large tradeoff between the number of individuals and the extent of phenotyping (for large phenotype networks there are likely a relatively small number of individuals).

The *naïve* Bayes model clearly outperformed logistic regression in this setting, showing that the integration of a large number of phenotypes with prior information has had a decidedly positive effect on the performance of the model. Yet, it is likely that the assumptions of *naïve* Bayes are detrimental to its overall performance. More sophisticated approaches, which are able to incorporate prior information as well as remove the assumption of independence among phenotypes, such as a DBN, are likely to yield superior models and improve stratification of those at risk of asthma and those who are not. Further, models that allow topological changes in the network, are likely to better reflect the pathogenesis of the disease.

### Areas of development

Harnessing the full power of a network-based approach raises several difficult issues. Perhaps the most basic is the comparison of networks describing corresponding data from different studies. For the large, hybrid networks envisaged here, the issue of replicating networks and quantifying their differences is a complex problem and one requiring further research. A second issue is the harmonization of clinical and biological measures between studies as well as the incorporation of disparate variable types, including continuous, discrete/binary data into the network. This complication alone requires advanced statistical methods to “augment” non-continuous variables (193). Asthma pathogenesis is further complicated by the existence of multiple asthma phenotypes, as described above. Since these different phenotypes are likely to follow differing paths of development, their corresponding networks are likely different and the interventions required to prevent them may also be different. An obvious approach to this problem is to construct different networks for each phenotype, but this approach would neglect the insight that might be gleaned from modeling all asthma phenotypes simultaneously within the same network. Finally, the inference of causal relationships from statistical data is a difficult and subtle task. There are some tools for causal inference, which can be adopted from other fields, such as Granger causality (194, 195); however, the limitations of causal inference from observational data alone should be emphasized and, ultimately, definitive demonstration of causality will require experimental interventions.

## Conclusion

Asthma is a complex disease with multiple factors acting in concert over long-time periods. Here, we have reviewed known asthma pathways, characterized the clear complexity of the asthma phenotype, and proposed that prospective birth cohorts offer an attractive way forward, via tracking of disease development and a broad range of patient-associated clinical, physiological, biochemical, immunological, and microbiological markers in large human populations over time. On the resultant datasets, statistical modeling that integrates the deep phenotyping/genetic profiling with expert human knowledge is likely to provide valuable insights for the next generation of studies into asthma’s pathogenesis.

## Conflict of Interest Statement

The authors declare that the research was conducted in the absence of any commercial or financial relationships that could be construed as a potential conflict of interest.

## Supplementary Material

The Supplementary Material for this article can be found online at http://www.frontiersin.org/Journal/10.3389/fimmu.2014.00447/abstract
