# Supplementary material for: Elucidation of Pathways Driving Asthma Pathogenesis: Development of a Systems-Level Analytic Strategy
Source: Front Immunol. 2014 Sep 23;5:447. doi: 10.3389/fimmu.2014.00447 (PMC4172064; doi:10.3389/fimmu.2014.00447)
Supplement: Supplementary file 1 [file Table_1.DOCX]

SUPPLEMENTARY TABLE S1

**Elucidation of pathways driving asthma pathogenesis: Development of a systems-level analytic strategy**

Michael L. Walker, Kathryn E. Holt, Gary P. Anderson, Shu Mei Teo, Peter D. Sly, Patrick G. Holt, Michael Inouye

**Phenotype Descriptions**

Number of Wheezy Lower Respiratory Tract Infections with HRV in last 12mnths at 1Y

Number of Lower Respiratory Tract Infections with RSV in last 12mnths at 1Y

Number of Severe Lower Respiratory Tract Infections with any virus in last 12mnths at 1Y

Number of Severe Lower Respiratory Tract Infections with multiple viruses in last 12mnths at 1Y

Number of Febrile Upper Respiratory Infections in last 12 mnths at 1Y

Number of Upper Respiratory Infections in last 12 mnths at 1Y

Number of Mild Lower Respiratory Infections in last 12 mnths at 1Y

Number of Severe Lower Respiratory Infections in last 12 mnths at 1Y

Number of Febrile Lower Respiratory Infections in last 12 mnths at 1Y

Number of Wheezy Lower Respiratory Infections in last 12 mnths at 1Y

Current wheeze (parent opinion) last 12 mnths at 1Y

Current wheeze (parent opinion) last 12 mnths at 2Y

Doctor diagnosed eczema last 12 mnths at 1Y

Doctor diagnosed eczema last 12 mnths at 2Y

Child exposed to cigarette smoke in last 12 months at 1 year

Attended childcare in last 12 months at 2 years of age (Yes/No)

Number of children under 16 years of age living in the home at 1 yr

Number of children under 16 years of age living in the home at 2 yrs

Number of children older than the proband living in the house at birth

Total protein concentration (g/l), mean of left and right breast, 6 months postpartum

25-Hydroxy Vitamin D (nmol/L) in mothers’s serum at 6 weeks postpartum

Omega-3 (n-3) essential polyunsaturated fatty acid content at 6 months of age expressed as weight % of total fatty acids

Omega-6 (n-6) essential polyunsaturated fatty acid content at 6 months of age expressed as weight % of total fatty acids

Omega-3 (n-3) essential polyunsaturated fatty acid content at 6 weeks of age expressed as weight % of total fatty acids

Omega-6 (n-6) essential polyunsaturated fatty acid content at 6 weeks of age expressed as weight % of total fatty acids

Log of number of copies/µg of DNA from CD4-Tcells in PBMC at 1 year

Child exposed to cat in the house, childcare or playgroup in last 12 months at 1 year (inside/outside/None)

Child exposed to cat in the house, childcare or playgroup in last 12 months at 2 years (inside/outside/None)

IgG1 to p4 at 2 years. P4 is a conserved outer membrane protein from H. influenzae

IgG1 to p6 at 2 years. P6 is a conserved outer membrane protein from H. influenza

IgG1 to PspA1 at 2 years. PspA1 is pneumococcal surface protein A family 1 of S. pneumonia

IgG1 to PspA2 at 2 years. PspA2 is pneumococcal surface protein A family 2 of S. pneumonia

IgG1 to PspC at 2 years. PspC is pneumococcal surface protein C of S. pneumonia

IgE to Staphylococcus aureus enterotoxinA at 2 years

Log10 transformed IgE to HDM at 2 years

Log10 transformed IgE to cat epithelium and dander at 2 years

Log10 transformed IgE to couch grass at 2 years

Log10 transformed IgE to peanut at 2 years

Log10 transformed IgG4 to HDM at 2 years

Log10 transformed IgG4 to cat at 2 years

Log10 transformed IgG4 to couch (bermuda) grass at 2 years

Log10 transformed IgG to Der p1 at 2 years

Log10 transformed IgG to rfel d1 at 2 years

Log10 transformed IgG to rPhl p1 at 1 year

Positive skin prick test to any of cat, rye grass, HDM, alternaria, aspergillus, cow’s milk or egg white at 2 years of age (True/False)
